# Artificial intelligence use patterns, attitudes, and psychological distress among medical students: a cross-sectional survey

**DOI:** 10.3389/fmed.2026.1965749

**Published:** 2026-10-22

**Authors:** Mesut Sari, Yasemin İmrek, Ali Evren Tufan

**Affiliations:** Department of Child and Adolescent Psychiatry, Bolu Abant İzzet Baysal University Faculty of Medicine, Bolu, Türkiye

**Keywords:** artificial intelligence, attitudes, DASS-21, medical education, medical students, online survey, psychological distress

## Abstract

**Background:**

Artificial intelligence (AI) tools are widely used by medical students, but associations of general use frequency and AI-related attitudes with psychological distress remain unclear.

**Methods:**

All 1,275 active students at one Turkish medical faculty were invited to an anonymous online survey conducted from 1 April to 1 May 2026; 249 of 251 respondents consented. Measures included general AI use, an investigator-translated 13-item Artificial Intelligence Attitude Scale (ATAI), and the validated Turkish Depression Anxiety Stress Scales-21. A general-distress composite was the primary outcome, and hierarchical regression used HC3 robust standard errors.

**Results:**

AI use was reported by 95.6% of participants, and 50.2% reported daily use. Mean doubled DASS-21 total was 56.97 (SD 31.55). The ATAI three-factor model showed mixed fit (CFI = .898, TLI = .872, RMSEA = .107, SRMR = .070). AI-frequency categories did not improve the adjusted model at the block level (change in *R*-squared = .016, *p* = .111; robust global *p* = .088). Adding exploratory ATAI factors increased R-squared by.052 (*p* = .002; robust global *p* = .009); Benefit was inversely, and Risk and Use positively, associated with distress. Subscale sensitivity analyses preserved coefficient directions, but only the inverse Benefit-Anxiety association survived correction across 15 additional focal tests (*q* = .032).

**Conclusion:**

AI use was nearly universal and served both educational and personal/non-educational purposes, but this sample did not provide stable evidence of an overall association between general use frequency and distress. ATAI associations are exploratory and may reflect measurement or common-method processes. Longitudinal multicenter studies should use formally adapted measures and objective indicators of AI engagement.

## Introduction

Artificial intelligence (AI) has moved rapidly into undergraduate medical education through generative language models, adaptive learning systems, virtual patients, automated feedback, and decision-support simulations (1–5). Recent reviews describe expanding use across self-directed study, formative assessment, information retrieval, and case-based learning, but they also emphasize heterogeneity in implementation and limited evidence about educational and psychological outcomes (1–3). Medical students commonly perceive AI as useful while simultaneously expressing concerns about accuracy, overreliance, privacy, ethics, and future professional roles (6).

Psychological distress is already common during medical education. Multi-institutional and international studies report substantial depressive, anxiety, and stress symptoms, although estimates vary with sampling, training context, workload, and measurement (7–10). AI could plausibly relate to distress in opposing ways. Rapid access to explanations and study support may increase perceived control, whereas uncertainty about reliability, professional displacement, data privacy, or dependence on AI may intensify threat appraisal. Cross-sectional self-report data cannot resolve these pathways, but careful characterization can identify associations for subsequent longitudinal investigation.

Emerging research on problematic or dependent AI use also cautions against assuming that AI engagement precedes psychological distress. In a two-wave cross-lagged study of adolescents, mental health problems predicted later AI dependence, whereas AI dependence did not predict later mental health problems (11). Among university students, academic stress was positively associated with AI dependence and statistically mediated part of the association between academic self-efficacy and dependence (12). These findings support reverse-causation and coping-based explanations, whereby students experiencing distress or academic pressure may engage more strongly with AI. However, this literature has focused on AI dependence or problematic use rather than general use frequency and attitudes among medical students.

Use frequency and attitudes should be distinguished. Frequency is a behavioral exposure with potentially restricted variability when uptake is nearly universal. Perceived benefits, risks, and willingness to engage are subjective appraisals that may relate to distress through shared affective and response processes. Accordingly, an association with an attitude score cannot be interpreted as an effect of AI use. Analyses should also avoid treating correlated depression, anxiety, and stress subscales as independent evidence for domain-specific effects (13).

We conducted an anonymous survey of students across all 6 years of a Turkish medical program and analyzed original data collected specifically for the ethics-approved project described below. The objectives were to (1) describe AI tools, frequency, duration, and purposes; (2) assess the internal consistency and structural fit of an investigator-translated Artificial Intelligence Attitude Scale (ATAI); (3) describe DASS-21 psychological distress; and (4) explore whether general AI-use frequency and ATAI factor scores were associated with a single general-distress outcome after adjustment for training year, sex, and psychiatric treatment history. We tested two non-directional analytical hypotheses: that AI-use frequency categories would contribute jointly to the adjusted model, and that the ATAI factor block would add explanatory information beyond frequency and covariates. Individual ATAI coefficients were exploratory. Because the analytic hypotheses and models were specified after data collection and were not preregistered, and because the ATAI wording had not undergone formal cross-cultural adaptation, all measurement and association analyses are presented as exploratory rather than confirmatory.

## Materials and methods

### Design and reporting framework

This was a single-center cross-sectional online survey. Reporting was guided by the STROBE statement and the Checklist for Reporting Results of Internet E-Surveys (CHERRIES) (14, 15). Data collection took place from 1 April to 1 May 2026. The survey window overlapped a scheduled Year 1 midterm period (20–24 April 2026); examination and rotation schedules differed across other years, and each participant's completion date relative to examinations was not analyzed.

### Dataset provenance and analytical status

The survey and dataset were created prospectively and exclusively for the project “Investigation of the relationships between artificial intelligence-assisted learning in medical education, clinical reasoning self-efficacy, and psychological well-being.” The present article reports the project's AI-use, AI-attitude, and psychological-distress components. No analyses based on this dataset have been published, accepted, or submitted in another manuscript, and the data have not been repurposed for a different study. This is not a secondary-data analysis. The analytic hypotheses and models were specified after data collection and were not preregistered; they are therefore treated as exploratory.

### Setting, participants, and recruitment

The eligible population comprised all 1,275 active students in Years 1–6 at Bolu Abant Izzet Baysal University Faculty of Medicine, Türkiye. Eligibility required active medical-student status and age of at least 18 years. All included respondents were adults (range 18–34 years). The responsible investigator sent individual invitations to the complete eligible population by SMS and/or WhatsApp. No reminder messages were sent, no financial or academic incentive was offered, and the investigator did not teach or assess the invited students.

The survey required sign-in to a Google Account and was configured to permit one response per account. Email addresses were not collected, and the research dataset contained no direct identifiers. Written informed consent was obtained electronically before participants accessed any study questions. Of the 251 individuals who reached the consent item, two declined and exited before any study question; the 249 consenting respondents constituted the original study dataset and the descriptive sample. No additional case selection was performed for this report. All retained scale items were complete, and no values were imputed. Branch-dependent AI questions were treated as not applicable for respondents who selected that they had never used an AI tool. Because the complete eligible population was invited, no *a priori* sample-size target was imposed; the achieved sample determined precision and was not intended to establish equivalence or exclude small associations.

### Ethics

The Bolu Abant Izzet Baysal University Non-Interventional Clinical Research Ethics Committee approved the prospective study protocol and the procedure for obtaining written informed consent electronically on 24 March 2026 (Decision No. 2026/104). Approval therefore preceded recruitment and the start of data collection on 1 April 2026. Participants consented to this study before completing any study item, and the data were not repurposed for a different project. The study was conducted in accordance with the Declaration of Helsinki. Participants received no payment.

### Measures

#### Sociodemographic and AI-use questionnaire

The questionnaire assessed age, sex, year of training, grade-point-average category, previous psychiatric treatment, current psychiatric treatment, previous AI use, commonly used AI tools, duration and general frequency of use, and multiple-response purposes. Six investigator-developed 1–5 agreement items asked about perceived learning quality, perceived support for clinical-reasoning practice, concern about physician replacement, concern about weakened critical thinking, perceived adequacy of AI training, and privacy/ethical concern. These six items were analyzed individually and not combined into a scale.

General AI frequency had five response levels: never, a few times per month, once per week, a few times per week, and daily. The gate question on previous use was treated as authoritative. For the primary model, sparse lower-frequency categories were combined into no use to once per week (*n* = 53), compared with a few times per week (*n* = 71) and daily use (*n* = 125). The original five categories and a monotonic ordinal score were retained as sensitivity specifications.

#### Artificial intelligence attitude scale

The 13-item Artificial Intelligence Attitude Scale (ATAI) was developed in Turkish pre-service teachers and comprises Benefit (items 1–3), Risk (items 4–7), and Use (items 8–13) factors, with responses from 1 (strongly disagree) to 5 (strongly agree) (16). It was selected *a priori* because the project examined attitudes toward AI broadly—not only generative AI in education—and because its brief three-domain structure distinguished perceived benefits, perceived risks, and willingness to use AI, which were conceptually relevant to the research question while limiting survey burden. A 13-item Turkish-English Generative Artificial Intelligence Attitude Scale for Students, organized into positive and negative attitude dimensions, was also available (17). That instrument is specifically framed around generative AI in educational settings; the broader scope and three separate domains of the ATAI were the reasons for the present choice. The ATAI was not originally validated in medical students. Subsequent 2026 studies have administered the source ATAI to Turkish health sciences, sports sciences, and nursing students (18–20), but those applications do not validate the wording administered here.

The source publication provides both Turkish and English forms. When assembling the online questionnaire, the responsible investigator was unaware that the published Turkish form was available and therefore translated the published English items into Turkish. The published Turkish form was identified subsequently. This was not a formal adaptation, and there was no psychometric rationale for modifying the published Turkish wording; using the source Turkish form unchanged would have been preferable. Comparison with the source Turkish form showed that 4 of 13 items (items 3–5 and 11) were identical and 9 items (items 1, 2, 6–10, 12, and 13) contained lexical or phrasing differences. Most differences involved near-synonymous wording, but semantic equivalence was not established. The source English item 12 omits the word “creative” that appears in the source Turkish form; the administered wording followed the English item and may therefore have been broader. Supplementary Table S6 provides an item-by-item comparison of the source English and Turkish forms and the administered wording. No independent forward/back-translation, expert-panel review, cognitive interview, source-author verification, or independent validation preceded data collection. Higher raw Risk scores indicated greater perceived risk; Risk items were not reverse-scored for factor-specific analyses. All factor-score analyses were exploratory and conditional on structural evaluation in this sample.

#### Depression anxiety stress scales-21

The DASS-21 assesses symptoms during the previous week using 21 items scored 0–3 (21). The validated adult Turkish form was used (22); a youth version was not required because all participants were at least 18 years old. Seven-item Depression, Anxiety, and Stress subscales were summed and multiplied by two for comparability with the DASS-42 metric. To reduce repeated testing of highly correlated self-reported outcomes, the primary association analysis used the doubled sum of all 21 items (range 0–126) as a single general-distress composite. This composite represents shared symptom burden, consistent with evidence for a general psychological-distress component alongside distinct Depression, Anxiety, and Stress dimensions (13). Its use does not imply that the DASS-21 is strictly unidimensional. The three subscales were also examined as separate outcomes in exploratory sensitivity analyses. Severity categories were descriptive screening indicators and were not interpreted as psychiatric diagnoses or population prevalence.

### Excluded questionnaire modules

The questionnaire also contained ten researcher-developed clinical task-confidence items and a ten-item perceived-stress module that did not reproduce the validated PSS-10 item set. These modules lacked an adequate basis for validated scoring and were excluded from every analysis in the present report. No CRESCAT instrument was administered, scored, or analyzed, and the study makes no claim to have measured objective clinical-reasoning performance or clinical-reasoning self-efficacy.

### Statistical analysis

Categorical data are reported as counts and percentages; continuous scores as means, standard deviations, medians, interquartile ranges, and ranges. Cronbach alpha and McDonald's omega from one-factor congeneric normal-theory maximum-likelihood models were calculated for retained scores. Maximum-likelihood confirmatory factor analyses of standardized item covariance matrices compared the proposed three-factor and one-factor structures for ATAI and DASS-21. Model evaluation included chi-square, CFI, TLI, RMSEA, SRMR, standardized loadings, and factor correlations. Given the ordinal items, modest sample, and absence of a formally adapted ATAI translation, these analyses were treated as exploratory structural checks rather than definitive validation.

Year-specific general-distress distributions were compared using a Kruskal–Wallis test with epsilon-squared effect size. The primary adjusted analysis was a three-block ordinary least-squares model with HC3 robust standard errors. Block 1 included year as a six-category variable, sex, and previous psychiatric treatment. Block 2 added the three-category AI-frequency variable. Block 3 added ATAI Benefit, Risk, and Use raw sums standardized within the complete-case regression sample. Each block was evaluated using conventional change-in-*F* tests and HC3 robust Wald *F* tests, with Block 1 compared against an intercept-only model. All three blocks used the same complete-case sample. Benjamini–Hochberg correction (23) was applied across the three ATAI coefficients as one exploratory family. No automated variable selection or imputation was used. One participant who preferred not to report sex was retained descriptively and excluded from adjusted models; the full model estimated 12 predictor degrees of freedom plus an intercept from 248 complete cases.

Diagnostics included residual inspection, Shapiro–Wilk and Breusch–Pagan tests, variance-inflation factors, leverage, and Cook distance. Sensitivity analyses used the original five frequency categories, an ordinal frequency trend, current rather than previous psychiatric treatment, and exclusion of observations with Cook distance greater than 4/*n*. Analyses used Python 3.12.14 with NumPy 2.3.5, pandas 2.2.3, SciPy 1.17.0, and openpyxl 3.1.5 for data import. The executable Python program calculated all reported estimates directly; companion SPSS syntax reproduces scoring and the primary model, including a MATRIX-based HC3 covariance calculation. Version-locked code, an analysis-variable codebook, an empty input template, and aggregate validation outputs are provided as Supplementary File 2; participant-level data are not included. Two-sided *p* < .05 was used, with adjusted q-values reported for the ATAI family. Supplementary Table S7 provides the complete analysis specification; S8 reports measurement-model fit, S9 reports the full three-block regression, and S10 reports the subscale sensitivity analyses.

Additional sensitivity analyses repeated the three-block specification for the doubled Depression, Anxiety, and Stress scores, using the same 248 complete cases, predictor coding, and ATAI standardization as the total-score model. Nominal HC3 *p*-values and 95% confidence intervals were reported. Benjamini–Hochberg *q*-values were also calculated across the 15 focal Block 3 tests (two AI-frequency contrasts and three ATAI coefficients for each of the three subscales) as a separate exploratory family; the original correction across the three ATAI coefficients in the total-score model was retained.

## Results

### Participants and AI-use patterns

The analytical sample comprised 249 students, corresponding to 19.5% of the eligible population. Mean age was 21.49 years (SD: 2.80; range: 18–34), 172 (69.1%) participants were women, 76 (30.5%) were men, and one (0.4%) preferred not to report sex. Representation varied by year, from 20 students in Year 4–69 in Year 2 (Table 1). Previous psychiatric treatment was reported by 62 (24.9%) and current treatment by 29 (11.6%).

**Table 1:** Participant characteristics and AI use (*N* = 249).

Characteristic	Category/statistic	*n* or value	%
Age, years	Mean (SD)	21.49 (2.80)	
Sex	Women	172	69.1
Sex	Men	76	30.5
Sex	Prefer not to report	1	0.4
Year of training	Year 1	65	26.1
Year of training	Year 2	69	27.7
Year of training	Year 3	32	12.9
Year of training	Year 4	20	8.0
Year of training	Year 5	26	10.4
Year of training	Year 6	37	14.9
Previous psychiatric treatment	Yes	62	24.9
Current psychiatric treatment	Yes	29	11.6
Previous AI use	Yes	238	95.6
AI frequency	No use to once/week	53	21.3
AI frequency	A few times/week	71	28.5
AI frequency	Daily	125	50.2

Percentages may not total 100 because of rounding. AI frequency categories used the previous-use gate question as authoritative.

AI use was reported by 238 students (95.6%). After the gate-question rule, 125 (50.2%) were classified as daily users, 71 (28.5%) as using AI a few times per week, and 53 (21.3%) as no use through once per week. Among the 238 users, 139 (58.4%) had used AI for more than 1 year. ChatGPT (80.3%) and Google Gemini (63.9%) were most frequently selected. Course study/review (84.5%), examination preparation (71.8%), medical terminology/concept learning (65.1%), and research/literature searching (62.6%) were common; 171 users (71.8%) also selected personal/non-educational use, and 38.2% selected clinical case or reasoning practice (Figure 1 and Supplementary Table S1).

**Figure 1:**
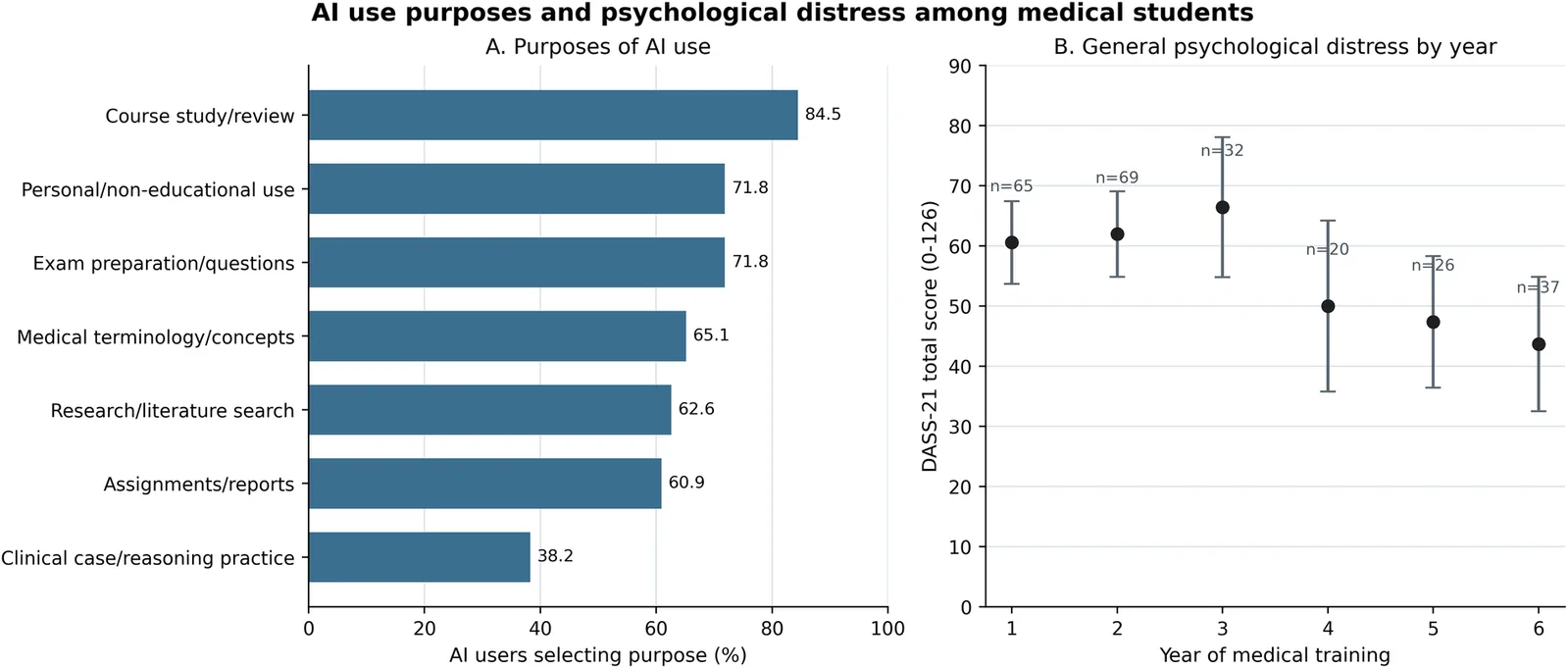
AI-use purposes and general psychological distress. **(A)** Multiple-response purposes among 238 respondents who reported prior AI use. **(B)** Independent year-cohort means and 95% confidence intervals for the DASS-21 general-distress composite. Points are intentionally not connected because the study was cross-sectional.

### Retained measures and measurement structure

Mean DASS-21 total score was 56.97 (SD: 31.55), with strong total-score internal consistency (alpha = .951; one-factor McDonald's omega = .951). The one-factor model yielded chi-square(189) = 766.04, *p* < .001, CFI = .828, TLI = .809, RMSEA = .111, and SRMR = .069. The proposed three-factor model yielded chi-square(186) = 631.03, *p* < .001, CFI = .867, TLI = .850, RMSEA = .098, and SRMR = .062 (Supplementary Table S8). The three-factor model fit better, but neither model showed strong absolute fit by conventional CFI/TLI and RMSEA benchmarks. Factor correlations ranged from .800 to .902, indicating substantial overlap without establishing strict unidimensionality. The total was therefore interpreted as a general-distress composite, with subscale sensitivity analyses used to examine whether association directions depended on the outcome dimension.

ATAI factor internal consistency ranged from alpha = .775 to .931 and McDonald's omega = .774 to .933 (Table 2). The proposed ATAI three-factor model substantially outperformed the one-factor model [chi-square(62) = 237.50 vs. chi-square(65) = 1,004.29; Supplementary Table S8], and standardized loadings ranged from.520 to.938. Absolute fit was mixed (CFI = .898, TLI = .872, RMSEA = .107, SRMR = .070), so the translated factor scores were retained only for exploratory analysis. Benefit and Use factors correlated .441; Risk correlations with the other factors were near zero.

**Table 2:** Descriptive statistics and internal consistency of retained scores.

Measure	Mean (SD)	Median (IQR)	Range	Alpha	Omega
DASS-21 total	56.97 (31.55)	58 (32–84)	0–126	0.951	0.951
DASS-21 Depression	19.47 (11.50)	20 (10–28)	0–42	0.885	0.885
DASS-21 Anxiety	16.10 (10.94)	16 (8–24)	0–42	0.868	0.869
DASS-21 Stress	21.40 (11.84)	22 (12–32)	0–42	0.906	0.907
ATAI Benefit	12.86 (2.48)	13 (12–15)	3–15	0.931	0.933
ATAI Risk	13.08 (3.76)	13 (10–16)	5–20	0.775	0.774
ATAI Use	19.37 (6.07)	20 (15–24)	6–30	0.870	0.870

DASS-21 subscales and total are doubled to the DASS-42 metric. ATAI scores derive from an investigator-translated form and are exploratory.

### General distress across training years

DASS-21 total scores differed across independent year cohorts [*H*(5) = 14.87, *p* = .011, epsilon^2^ = .041]. Mean scores were 60.55, 61.97, 66.44, 50.00, 47.38, and 43.68 for Years 1–6, respectively (Figure 1). These are between-cohort differences observed at one time point and do not represent individual trajectories.

### Hierarchical model of general psychological distress

Block 1 (training year, sex, and previous psychiatric treatment) explained 10.6% of DASS-21 total variance (*R*^2^ = .106; adjusted *R*^2^ = .080). Relative to the intercept-only model, Delta *R*^2^ = .106, *F*change(7, 240) = 4.06, *p* < .001, and the HC3 robust test was *F*(7, 240) = 3.67, *p* < .001. Adding three-category AI frequency in Block 2 yielded *R*^2^ = .122 and adjusted *R*^2^ = .089. The increase in *R*^2^ was.016 and was not statistically significant [*F*change(2, 238) = 2.22, *p* = .111]; the HC3 global frequency test was also nonsignificant [robust *F*(2, 238) = 2.45, *p* = .088]. Consequently, nominal coefficients for a few times per week and daily use were not interpreted as evidence of category-specific effects.

Adding the three exploratory ATAI factors increased *R*^2^ by.052 [*F*change(3, 235) = 4.98, *p* = .002; robust *F*(3, 235) = 3.94, *p* = .009], yielding total *R*^2^ = .175 and adjusted *R*^2^ = .133. Per one-SD higher factor score, Benefit was associated with lower general distress (*B* = −4.90, 95% CI: −9.14 to −0.65; *q* = .036), whereas Risk (*B* = 4.98, 95% CI: 0.87–9.09; *q* = .036) and Use (*B* = 4.69, 95% CI: 0.26–9.12; *q* = .038) were associated with higher distress. Training year was significant as a block (robust *p* = .043), with Year 6 lower than Year 1 in the full model; these estimates describe cohort differences rather than change over time. Table 3 reports the final Block 3 coefficients. Supplementary Table S9 provides the coefficients for all three blocks, together with *R*^2^, adjusted *R*^2^, change in *R*^2^, conventional *F*-change tests, and HC3 robust tests at each step.

**Table 3:** Final block of the hierarchical model for DASS-21 total score (*n* = 248).

Term	*B*	HC3 SE	95% CI	*p*	*q*
Year 2 vs. Year 1	−0.15	4.88	−9.77, 9.48	0.976	
Year 3 vs. Year 1	1.95	6.96	−11.76, 15.67	0.779	
Year 4 vs. Year 1	−13.34	8.64	−30.36, 3.68	0.124	
Year 5 vs. Year 1	−12.94	6.98	−26.70, 0.81	0.065	
Year 6 vs. Year 1	−16.79	6.88	−30.35, −3.23	0.015	
Men vs. women	−7.53	4.35	−16.09, 1.04	0.085	
Previous psychiatric treatment	10.04	5.13	−0.06, 20.15	0.051	
AI a few times/week	11.99	5.59	0.98, 23.00	0.033	
AI daily	10.46	5.30	0.02, 20.90	0.050	
ATAI Benefit (per SD)	−4.90	2.15	−9.14, −0.65	0.024	0.036
ATAI Risk (per SD)	4.98	2.09	0.87, 9.09	0.018	0.036
ATAI Use (per SD)	4.69	2.25	0.26, 9.12	0.038	0.038

Reference groups: Year 1, women, no previous psychiatric treatment, and AI use no more than once per week. ATAI scores were standardized. Benjamini-Hochberg *q*-values apply only to the three ATAI coefficients. Overall model *R*^2^ = .175; adjusted *R*^2^ = .133; maximum VIF = 2.27.

### Diagnostics and sensitivity analyses

Residual normality was not rejected (Shapiro–Wilk *W* = .990, *p* = .073), and there was no evidence of heteroscedasticity by the Breusch–Pagan test (*p* = .414); HC3 standard errors were nevertheless retained. Maximum VIF was 2.27. Twelve observations exceeded Cook *D* > 4/*n* and three exceeded leverage > 2*p*/*n*. Excluding the Cook-distance observations did not reverse ATAI coefficient directions and strengthened their estimates. With the original five AI-frequency categories, individual frequency coefficients were imprecise and nonsignificant; the ordinal trend estimate was *B* = 3.24 per category (95% CI: −0.17 to 6.65; *p* = .062). Replacing previous with current psychiatric treatment did not materially change ATAI directions (Supplementary Table S4).

Subscale sensitivity models retained all 248 complete cases. The added AI-frequency block was nonsignificant for Depression [robust *F*(2, 238) = 2.30, *p* = .102], Anxiety [robust *F*(2, 238) = 1.90, *p* = .152], and Stress [robust *F*(2, 238) = 1.89, *p* = .154]. In the final models, both frequency contrasts were positive across subscales; ATAI Benefit was negative, and Risk and Use were positive, for all three outcomes. Statistical support nevertheless varied by subscale. After correction across the 15 additional focal tests, only the inverse Benefit-Anxiety association remained below *q* = .05 (*B* = −2.39, 95% CI: −3.91 to −0.87; nominal *p* = .002; *q* = .032). Supplementary Table S10 presents the subscale estimates, HC3 standard errors, confidence intervals, nominal *p*-values, additional *q*-values, and model tests. These analyses were exploratory.

## Discussion

In this single-center sample, AI use was nearly universal and served both educational and non-educational purposes. More than four fifths of users selected course study or review, while 71.8% also selected personal/non-educational use. Half of participants reported daily use. Nevertheless, general use frequency did not produce a statistically supported block-level improvement in the adjusted distress model, and its estimate depended on category specification. The appropriate interpretation is not that AI frequency is unrelated to distress, but that this sample did not provide stable evidence for an overall frequency association.

The frequency result should be considered in light of substantial range restriction. Nearly four fifths of the sample used AI a few times per week or daily, leaving limited contrast at the lower end. General frequency also did not quantify learning quality, duration per session, task complexity, verification behavior, or reliance. Similar frequency values may therefore represent very different forms of engagement. Future work should use behavioral logs or purpose-specific exposure measures rather than assuming equal information across verbal frequency categories.

The exploratory ATAI block added explanatory variance: perceived Benefit was inversely associated with general distress, while Risk and Use were positively associated. These directions may reflect perceived control, threat appraisal, or greater cognitive and emotional engagement with AI. They could also arise from negative affectivity, response consistency, or other common-method processes because attitudes and symptoms were measured by self-report in the same session. The positive Use coefficient, despite the inverse Benefit coefficient, particularly cautions against a simple beneficial-vs.-harmful interpretation. Reverse causation is plausible: a two-wave adolescent study found that mental health problems predicted later AI dependence rather than the reverse (11), and a cross-sectional university study linked academic stress with greater AI dependence (12). The positive Use coefficient may therefore reflect distressed or academically pressured students being more willing to engage with AI, rather than an effect of willingness to use AI on distress. The present data do not show that AI attitudes cause distress or that changing attitudes would improve wellbeing.

Although the subscale sensitivity analyses retained the coefficient directions, statistical support was uneven and only the inverse Benefit-Anxiety association survived correction across the 15 additional focal tests. These analyses examine related outcomes in the same participants; they do not provide independent replication or resolve uncertainty about the translated ATAI constructs.

Measurement results further limit inference. Although ATAI subscales had acceptable to excellent internal consistency and the proposed three-factor structure clearly outperformed one factor, absolute CFA fit was not strong. Selection of a broad, three-domain AI-attitude instrument was conceptually aligned with this study, but the administered Turkish wording was produced by one investigator from the English form without formal cross-cultural adaptation. Most differences from the source Turkish form were lexical, yet the item 12 difference between the source language forms and the administered wording could have broadened its content. Recent use of the source ATAI in Turkish student samples (18–20) does not validate the modified wording used here. Accordingly, the scores may not reproduce the intended constructs in Turkish medical students, and the observed associations should generate hypotheses rather than establish construct-specific effects. A formal adaptation should include permission and source verification, independent forward and backward translation, expert review, cognitive interviews, and validation in an independent sample; studies focused specifically on generative AI in education could also consider the student scale developed for that construct (17).

General distress differed across training-year cohorts, with lower adjusted scores in Year 6 than Year 1. These data do not demonstrate improvement over time because different cohorts were assessed once. The survey overlapped a Year 1 midterm period, while examination and rotation schedules differed across the remaining years; individual assessment proximity and workload were not measured. These timing differences, cohort effects, and response selection could contribute to the observed year pattern.

DASS symptom levels were high, consistent with longstanding concern about medical-student wellbeing (7–10). However, DASS-21 categories are screening descriptors rather than diagnoses, and the voluntary response proportion was 19.5%. Students experiencing distress or holding strong AI views may have been more likely to participate. The estimates therefore should not be interpreted as population prevalence.

### Strengths and limitations

Strengths include invitation of the full student population, inclusion of all 6 training years, explicit reporting of recruitment and duplicate-response controls, complete retained scale data, separation of general AI use from purposes, use of a single general-distress primary outcome, formal block tests, robust standard errors, and multiple sensitivity specifications. The revised figure presents independent cohorts without implying longitudinal progression.

Limitations are substantial. The study was cross-sectional, single-center, voluntary, and entirely self-reported; causal direction cannot be determined. All principal variables were collected in the same online session, and no marker variable, procedural separation, behavioral measure, observer rating, or external comparison group was available. Common negative affectivity, threat appraisal, response style, or consistency could therefore contribute to associations between ATAI and distress, and the data cannot establish construct-specific effects. Fresh graduates or students from a non-AI comparison setting were not recruited. Participation was low and class representation was uneven, while sex distribution for the eligible population was unavailable. The relatively high proportion reporting previous psychiatric treatment (24.9%) may also indicate self-selection by students with greater mental-health concerns and may partly account for the high DASS-21 scores and observed associations. AI uptake was concentrated at the upper end, and frequency did not measure educational intensity or quality. ATAI wording was not formally adapted, was not validated for medical students, and showed limited absolute factor-model fit; measurement invariance across training stages was not tested. CFA used covariance-based maximum likelihood for ordinal items and was exploratory. DASS-21 factor-model fit was also limited, and using the total as a composite does not establish strict unidimensionality. The analytic hypotheses and models were developed after data collection and were not preregistered; all inferential findings are exploratory and hypothesis-generating. One participant was excluded from adjusted models because sex was not reported. Academic workload and assessment timing were not measured at the individual level.

### Implications

Medical schools should not infer psychological benefit or harm from use frequency alone. Multicenter longitudinal designs should combine formally adapted attitude instruments, validated well-being measures, objective or diary-based AI exposure, task-specific educational uses, and assessment timing. Qualitative work may clarify why active engagement can coexist with perceived benefit, risk, and distress. Educational programs should evaluate AI literacy, verification practices, privacy, and critical use directly rather than presuming that greater exposure is inherently advantageous.

## Conclusion

AI use was widespread and encompassed both educational and personal/non-educational purposes among medical students in this survey. This sample did not provide stable block-level evidence of an overall association between general use frequency and psychological distress, and restricted variability limited inference about small effects. Investigator-translated AI attitude factors showed exploratory associations with distress, but limited structural fit, lack of formal adaptation, and common-method processes preclude construct-specific conclusions. Coefficient directions were similar across the DASS-21 subscales, but statistical support was uneven. More rigorous longitudinal research with validated translations and objective measures of AI engagement is needed.

## Data Availability

The raw data supporting the conclusions of this article will be made available by the authors, without undue reservation.
