# Supplementary material for: Artificial intelligence use patterns, attitudes, and psychological distress among medical students: a cross-sectional survey
Source: Front Med (Lausanne). 2026 Oct 22;13:1965749. doi: 10.3389/fmed.2026.1965749 (PMC13735594; doi:10.3389/fmed.2026.1965749)
Supplement: Supplementary file 1 [file Table1.docx]

Supplementary Material — Tables S1-S10

Artificial intelligence use patterns, attitudes, and psychological distress among medical students: a cross-sectional survey

# Supplementary Table S1. AI-use tools, duration, and purposes

| **Domain** | **Category** | **n** | **% of users** |
| --- | --- | --- | --- |
| Duration among AI users | More than 1 year | 139 | 58.4 |
| Duration among AI users | 6-12 months | 63 | 26.5 |
| Duration among AI users | 1-6 months | 31 | 13.0 |
| Duration among AI users | Less than 1 month | 5 | 2.1 |
| Tool among AI users | ChatGPT | 191 | 80.3 |
| Tool among AI users | Google Gemini | 152 | 63.9 |
| Tool among AI users | Microsoft Copilot | 5 | 2.1 |
| Tool among AI users | Claude | 20 | 8.4 |
| Tool among AI users | NotebookLM | 1 | 0.4 |
| Tool among AI users | Grok | 1 | 0.4 |
| Purpose among AI users | Course study / topic review | 201 | 84.5 |
| Purpose among AI users | Clinical case analysis / clinical reasoning practice | 91 | 38.2 |
| Purpose among AI users | Assignment / report preparation | 145 | 60.9 |
| Purpose among AI users | Exam preparation / question practice | 171 | 71.8 |
| Purpose among AI users | Medical terminology / concept learning | 155 | 65.1 |
| Purpose among AI users | Research / literature search | 149 | 62.6 |
| Purpose among AI users | Personal use (non-educational) | 171 | 71.8 |

Multiple tools and purposes could be selected; percentages therefore do not sum to 100. Denominator = 238 AI users.

# Supplementary Table S2. Investigator-developed AI opinion items

| **Item (1=strongly disagree, 5=strongly agree)** | **Mean** | **SD** | **Median** | **IQR** |
| --- | --- | --- | --- | --- |
| a) AI tools improve the quality of my learning in medical education. | 4.14 | 1.02 | 4 | 4-5 |
| b) AI tools help me develop my clinical reasoning skills. | 3.71 | 1.15 | 4 | 3-5 |
| c) I am concerned that AI will replace physicians in the future. | 2.36 | 1.23 | 2 | 1-3 |
| d) Overreliance on AI tools may weaken my critical-thinking skills. | 3.80 | 1.13 | 4 | 3-5 |
| e) I believe I have received adequate training on the use of AI tools in medical education. | 2.69 | 1.19 | 3 | 2-3 |
| f) I am concerned about data privacy and ethical issues when using AI tools. | 3.45 | 1.32 | 4 | 2-5 |

Items were administered in Turkish; English translations are shown. Items were analyzed individually and were not combined into a score.

# Supplementary Table S3. DASS-21 symptom-severity screening categories

| **Subscale** | **Screening category** | **n** | **%** |
| --- | --- | --- | --- |
| Depression | Normal | 52 | 20.9 |
| Depression | Mild | 34 | 13.7 |
| Depression | Moderate | 53 | 21.3 |
| Depression | Severe | 38 | 15.3 |
| Depression | Extremely severe | 72 | 28.9 |
| Anxiety | Normal | 62 | 24.9 |
| Anxiety | Mild | 14 | 5.6 |
| Anxiety | Moderate | 42 | 16.9 |
| Anxiety | Severe | 36 | 14.5 |
| Anxiety | Extremely severe | 95 | 38.2 |
| Stress | Normal | 76 | 30.5 |
| Stress | Mild | 37 | 14.9 |
| Stress | Moderate | 33 | 13.3 |
| Stress | Severe | 54 | 21.7 |
| Stress | Extremely severe | 49 | 19.7 |

Categories describe symptom-severity screening scores and must not be interpreted as psychiatric diagnoses or population prevalence.

# Supplementary Table S4. Sensitivity analyses for DASS-21 total

| **Specification** | **Term** | **B** | **HC3 SE** | **95% CI** | **p** | **n** |
| --- | --- | --- | --- | --- | --- | --- |
| Five-category frequency | AI frequency: a few times/month | -10.19 | 10.32 | -30.52, 10.14 | 0.324 | 248 |
| Five-category frequency | AI frequency: once/week | -0.10 | 10.09 | -19.97, 19.78 | 0.992 | 248 |
| Five-category frequency | AI frequency: a few times/week | 7.57 | 8.60 | -9.38, 24.52 | 0.380 | 248 |
| Five-category frequency | AI frequency: daily | 6.06 | 8.44 | -10.57, 22.68 | 0.474 | 248 |
| Five-category frequency | ATAI Benefit | -4.82 | 2.19 | -9.13, -0.52 | 0.028 | 248 |
| Five-category frequency | ATAI Risk | 4.85 | 2.10 | 0.72, 8.98 | 0.022 | 248 |
| Five-category frequency | ATAI Use | 4.41 | 2.28 | -0.07, 8.90 | 0.054 | 248 |
| Ordinal frequency trend | AI frequency score | 3.24 | 1.73 | -0.17, 6.65 | 0.062 | 248 |
| Ordinal frequency trend | ATAI Benefit | -5.03 | 2.15 | -9.26, -0.79 | 0.020 | 248 |
| Ordinal frequency trend | ATAI Risk | 4.76 | 2.11 | 0.60, 8.92 | 0.025 | 248 |
| Ordinal frequency trend | ATAI Use | 4.80 | 2.27 | 0.33, 9.28 | 0.035 | 248 |
| Current treatment instead of history | AI frequency: a few times/week | 12.54 | 5.60 | 1.50, 23.57 | 0.026 | 248 |
| Current treatment instead of history | AI frequency: daily | 9.94 | 5.39 | -0.68, 20.56 | 0.066 | 248 |
| Current treatment instead of history | ATAI Benefit | -4.76 | 2.18 | -9.06, -0.46 | 0.030 | 248 |
| Current treatment instead of history | ATAI Risk | 4.60 | 2.14 | 0.38, 8.82 | 0.033 | 248 |
| Current treatment instead of history | ATAI Use | 4.99 | 2.13 | 0.78, 9.19 | 0.020 | 248 |
| Current treatment instead of history | Current psychiatric treatment | 18.72 | 7.09 | 4.75, 32.69 | 0.009 | 248 |
| Exclude Cook D > 4/n | AI frequency: a few times/week | 9.75 | 5.35 | -0.79, 20.28 | 0.070 | 236 |
| Exclude Cook D > 4/n | AI frequency: daily | 9.68 | 5.05 | -0.27, 19.62 | 0.056 | 236 |
| Exclude Cook D > 4/n | ATAI Benefit | -7.23 | 2.01 | -11.20, -3.27 | <.001 | 236 |
| Exclude Cook D > 4/n | ATAI Risk | 6.43 | 1.82 | 2.85, 10.01 | <.001 | 236 |
| Exclude Cook D > 4/n | ATAI Use | 5.88 | 2.00 | 1.94, 9.82 | 0.004 | 236 |

Sensitivity analyses were exploratory. The Cook-distance analysis excluded observations with Cook D>4/n.

# Supplementary Table S5. Publicly available class distribution comparison

| **Year** | **Publicly reported population** | **Sample n** | **Approximate response %** |
| --- | --- | --- | --- |
| Year 1 | 280 | 65 | 23.2 |
| Year 2 | 234 | 69 | 29.5 |
| Year 3 | 218 | 32 | 14.7 |
| Year 4 | 192 | 20 | 10.4 |
| Year 5 | 199 | 26 | 13.1 |
| Year 6 | 152 | 37 | 24.3 |

Population figures are from the BAIBÜ Faculty of Medicine 2024-2025 activity report and are not temporally matched to the 2025-2026 survey. They are provided only as contextual information, not as a formal nonresponse analysis.

# Supplementary Table S6. Item-level summary of ATAI source-form and administered-wording differences

| **Item / factor** | **Source English vs source Turkish** | **Administered vs source Turkish** | **Classification** | **Interpretation** |
| --- | --- | --- | --- | --- |
| 1 / Benefit | English “advancement”; source Turkish “gelişme.” | “gelişme” → “ilerleme” | Lexical | Near-synonymous; the administered term follows the English wording more literally. |
| 2 / Benefit | English uses “believe”; source Turkish uses “düşünüyorum.” | “yaşam” → “hayat”; “düşünüyorum” → “inanıyorum” | Lexical and stance verb | The life terms are near-synonymous; “inanıyorum” follows English “believe.” |
| 3 / Benefit | Semantically aligned. | No difference. | Exact | The administered wording matches the source Turkish form. |
| 4 / Risk | Semantically aligned. | No difference. | Exact | The administered wording matches the source Turkish form. |
| 5 / Risk | Semantically aligned. | No difference. | Exact | The administered wording matches the source Turkish form. |
| 6 / Risk | English “human labor”; source Turkish “insan gücü.” | “insan gücü” → “insan iş gücü”; “endişe ediyorum” → “endişe duyuyorum” | Lexical | ‘İş gücü’ follows English ‘labor’ and may be narrower than ‘insan gücü’; the concern verbs are near-synonymous. |
| 7 / Risk | English uses “believe”; source Turkish uses “düşünüyorum.” | “düşünüyorum” → “inanıyorum” | Stance verb | The administered stance verb follows the English wording; equivalence was not tested. |
| 8 / Use | English “enjoy”; source Turkish uses “seviyorum.” | “metinsel içerik” → “metin içeriği”; “kullanmayı seviyorum” → “kullanmaktan keyif alıyorum” | Lexical and phrasing | Close lexical/phrasing correspondence; equivalence was not tested. |
| 9 / Use | Semantically aligned. | “konusundaki” → “alanındaki” | Lexical | Near-synonymous in context. |
| 10 / Use | English “enjoy”; source Turkish uses “hoşlanıyorum.” | “konularda” → “konular hakkında”; “hoşlanıyorum” → “keyif alıyorum” | Lexical and phrasing | Close lexical/phrasing correspondence; equivalence was not tested. |
| 11 / Use | Semantically aligned. | No difference. | Exact | The administered wording matches the source Turkish form. |
| 12 / Use | Source English omits “creative”; source Turkish includes “yaratıcı.” | “yaratıcı” omitted; wording otherwise paraphrased. | Potentially broader | The administered wording follows the English form and may cover visual products beyond explicitly creative products. |
| 13 / Use | Semantically aligned. | Function word “olarak” omitted. | Lexical | Function-word difference; semantic equivalence was not tested. |

Note. Source-form comparisons are based on Aktay et al. (reference 16 in the main manuscript), and the administered wording was verified against the survey dataset headings. Four of 13 items (3–5 and 11) were identical; nine (1, 2, 6–10, 12, and 13) contained lexical or phrasing differences. The English item 12 omits the word ‘creative’ present in the source Turkish form; the administered wording followed the English form and may therefore be broader. Only brief lexical contrasts are reported; the complete source instrument is not reproduced. This comparison was conducted after peer review and is a transparency exercise, not a retrospective validation or back-translation. Semantic equivalence and measurement equivalence were not established.

# Supplementary Table S7. Complete analysis specification for reproducibility

| **Component** | **Specification** |
| --- | --- |
| Design and source | Prospective, single-center cross-sectional online survey created exclusively for the ethics-approved project; data collected 1 April–1 May 2026 at Bolu Abant Izzet Baysal University Faculty of Medicine. |
| Sample flow | 1,275 eligible adults invited; 251 reached the consent item; 2 declined before study questions; 249 formed the dataset and descriptive sample; 248 complete cases entered adjusted models because 1 participant preferred not to report sex. |
| Missing data | No retained scale items were missing and no imputation was used. Branch-dependent AI-use items were not applicable to respondents who reported never using AI. |
| Primary outcome | DASS-21 general-distress total = 2 × sum of all 21 item scores; range 0–126. Separate doubled Depression, Anxiety, and Stress totals (range 0–42 each) were also examined in exploratory sensitivity models. Severity categories remained descriptive. |
| ATAI scores | Benefit = sum of items 1–3; Risk = sum of items 4–7 without reverse scoring; Use = sum of items 8–13. Each raw sum was standardized using the mean and sample SD (ddof=1) within the n=248 regression sample; the same standardized values were used for all outcomes. |
| Block 1 | Training year as five indicators (Year 1 reference), sex (women reference), and previous psychiatric treatment (no reference). |
| Block 2 | AI frequency as two indicators: a few times/week and daily; no use through once/week was the reference category. |
| Block 3 | Standardized ATAI Benefit, Risk, and Use scores. |
| Inference | OLS coefficients with HC3 standard errors, t-based p-values and 95% CIs; conventional F-change tests and HC3 robust Wald F tests at every step, with Block 1 compared against an intercept-only model. Original Benjamini–Hochberg correction across the three total-score ATAI coefficients. |
| Diagnostics | Residual inspection, Shapiro–Wilk test, Breusch–Pagan test, VIF, leverage, and Cook distance. |
| Sensitivity analyses | Original five frequency categories; ordinal frequency trend; current rather than previous psychiatric treatment; exclusion of observations with Cook D > 4/n; separate Depression, Anxiety and Stress outcomes using the same complete cases and predictor coding. |
| Software | Python 3.12.14; NumPy 2.3.5; pandas 2.2.3; SciPy 1.17.0; openpyxl 3.1.5. Companion SPSS syntax includes a MATRIX-based HC3 calculation for the primary model. |
| Code and data | Version-locked code, codebook, empty input template, and aggregate validation outputs are in Supplementary File 2. Participant-level data are not included; access follows the manuscript data-availability statement. |
| Additional multiplicity correction | A separate exploratory BH family comprised 15 final-model focal tests: two AI-frequency contrasts and three ATAI coefficients for each of the three DASS-21 subscales. Nominal p-values and CIs are shown alongside the additional q-values; the original three-test family was retained. |
| Analytical status | Hypotheses and analytic models were developed after data collection and were not preregistered. All measurement and association analyses are exploratory and hypothesis-generating. |

# Supplementary Table S8. Maximum-likelihood measurement-model fit

| **Model** | **n** | **χ²** | **df** | **p** | **CFI** | **TLI** | **RMSEA** | **SRMR** |
| --- | --- | --- | --- | --- | --- | --- | --- | --- |
| ATAI three-factor | 249 | 237.50 | 62 | <.001 | .898 | .872 | .107 | .070 |
| ATAI one-factor | 249 | 1004.29 | 65 | <.001 | .456 | .347 | .241 | .179 |
| DASS-21 three-factor | 249 | 631.03 | 186 | <.001 | .867 | .850 | .098 | .062 |
| DASS-21 one-factor | 249 | 766.04 | 189 | <.001 | .828 | .809 | .111 | .069 |

Note. Models used standardized item covariance matrices and normal-theory maximum likelihood. Because the items are ordinal and the ATAI wording was not formally adapted, these are exploratory structural checks rather than validation analyses.

# Supplementary Table S9. Full three-block hierarchical regression for DASS-21 total

**A. Coefficients at each step (n=248)**

**Block 1 — training year, sex and previous psychiatric treatment**

| **Term** | **B** | **HC3 SE** | **95% CI** | **p** | **q** |
| --- | --- | --- | --- | --- | --- |
| Intercept | 62.39 | 3.82 | 54.87, 69.90 | <.001 | — |
| Year 2 vs Year 1 | -1.00 | 5.02 | -10.89, 8.90 | 0.843 | — |
| Year 3 vs Year 1 | 2.45 | 6.87 | -11.07, 15.97 | 0.722 | — |
| Year 4 vs Year 1 | -14.00 | 8.72 | -31.17, 3.17 | 0.110 | — |
| Year 5 vs Year 1 | -17.03 | 6.60 | -30.02, -4.04 | 0.010 | — |
| Year 6 vs Year 1 | -21.60 | 6.73 | -34.85, -8.35 | 0.001 | — |
| Men vs women | -8.63 | 4.30 | -17.10, -0.16 | 0.046 | — |
| Previous psychiatric treatment | 12.57 | 5.22 | 2.29, 22.85 | 0.017 | — |

**Block 2 — covariates plus AI frequency**

| **Term** | **B** | **HC3 SE** | **95% CI** | **p** | **q** |
| --- | --- | --- | --- | --- | --- |
| Intercept | 53.81 | 5.26 | 43.46, 64.17 | <.001 | — |
| Year 2 vs Year 1 | -0.83 | 5.08 | -10.83, 9.17 | 0.870 | — |
| Year 3 vs Year 1 | 2.10 | 6.97 | -11.63, 15.84 | 0.763 | — |
| Year 4 vs Year 1 | -14.35 | 8.70 | -31.49, 2.80 | 0.101 | — |
| Year 5 vs Year 1 | -16.16 | 6.56 | -29.09, -3.23 | 0.015 | — |
| Year 6 vs Year 1 | -18.23 | 6.70 | -31.43, -5.02 | 0.007 | — |
| Men vs women | -8.06 | 4.32 | -16.58, 0.45 | 0.063 | — |
| Previous psychiatric treatment | 11.25 | 5.17 | 1.06, 21.44 | 0.031 | — |
| AI a few times/week | 10.93 | 5.53 | 0.03, 21.83 | 0.049 | — |
| AI daily | 9.98 | 4.99 | 0.16, 19.81 | 0.047 | — |

**Block 3 — covariates, AI frequency and exploratory ATAI factors**

| **Term** | **B** | **HC3 SE** | **95% CI** | **p** | **q** |
| --- | --- | --- | --- | --- | --- |
| Intercept | 52.61 | 5.28 | 42.21, 63.00 | <.001 | — |
| Year 2 vs Year 1 | -0.15 | 4.88 | -9.77, 9.48 | 0.976 | — |
| Year 3 vs Year 1 | 1.95 | 6.96 | -11.76, 15.67 | 0.779 | — |
| Year 4 vs Year 1 | -13.34 | 8.64 | -30.36, 3.68 | 0.124 | — |
| Year 5 vs Year 1 | -12.94 | 6.98 | -26.70, 0.81 | 0.065 | — |
| Year 6 vs Year 1 | -16.79 | 6.88 | -30.35, -3.23 | 0.015 | — |
| Men vs women | -7.53 | 4.35 | -16.09, 1.04 | 0.085 | — |
| Previous psychiatric treatment | 10.04 | 5.13 | -0.06, 20.15 | 0.051 | — |
| AI a few times/week | 11.99 | 5.59 | 0.98, 23.00 | 0.033 | — |
| AI daily | 10.46 | 5.30 | 0.02, 20.90 | 0.050 | — |
| ATAI Benefit (per SD) | -4.90 | 2.15 | -9.14, -0.65 | 0.024 | 0.036 |
| ATAI Risk (per SD) | 4.98 | 2.09 | 0.87, 9.09 | 0.018 | 0.036 |
| ATAI Use (per SD) | 4.69 | 2.25 | 0.26, 9.12 | 0.038 | 0.038 |

Note. Reference categories are Year 1, women, no previous psychiatric treatment, and AI use no more than once per week. ATAI raw sums were standardized within the common n=248 sample. Confidence intervals and nominal p-values use HC3 standard errors with residual degrees of freedom 240, 238 and 235 for Blocks 1, 2 and 3, respectively. q-values apply only to the three ATAI coefficients in Block 3; a dash indicates that this correction is not applicable. All estimates are exploratory.

# Supplementary Table S9 (continued). B. Model fit and tests at each step

| **Block** | **R²** | **Adjusted R²** | **Change in R²** | **F-change (df₁,df₂)** | **p** | **HC3 added-block F (df₁,df₂)** | **p** | **HC3 overall F (df₁,df₂)** | **p** |
| --- | --- | --- | --- | --- | --- | --- | --- | --- | --- |
| 1 | 0.106 | 0.080 | 0.106 | 4.06 (7,240) | <.001 | 3.67 (7,240) | <.001 | 3.67 (7,240) | <.001 |
| 2 | 0.122 | 0.089 | 0.016 | 2.22 (2,238) | 0.111 | 2.45 (2,238) | 0.088 | 3.49 (9,238) | <.001 |
| 3 | 0.175 | 0.133 | 0.052 | 4.98 (3,235) | 0.002 | 3.94 (3,235) | 0.009 | 3.72 (12,235) | <.001 |

Note. All models use the same 248 complete cases. Block 1 is compared against an intercept-only model; Block 2 adds the two AI-frequency indicators and Block 3 adds the three standardized ATAI scores. F-change tests are conventional OLS tests. HC3 added-block tests jointly test the newly entered coefficients, while HC3 overall tests jointly test all non-intercept coefficients in the corresponding model. Parentheses give numerator and denominator degrees of freedom. R² values are descriptive and are not robust-adjusted effect measures.

# Supplementary Table S10. Exploratory separate-outcome DASS-21 sensitivity analyses

**A. Focal coefficients in the final three-block models (n=248)**

| **Outcome** | **Term** | **B** | **HC3 SE** | **95% CI** | **p** | **Additional q** |
| --- | --- | --- | --- | --- | --- | --- |
| Depression | AI a few times/week | 4.60 | 2.10 | 0.47, 8.74 | 0.029 | 0.081 |
| Depression | AI daily | 4.00 | 1.95 | 0.15, 7.85 | 0.042 | 0.081 |
| Depression | ATAI Benefit (per SD) | -1.43 | 0.80 | -3.01, 0.14 | 0.074 | 0.100 |
| Depression | ATAI Risk (per SD) | 2.01 | 0.78 | 0.46, 3.55 | 0.011 | 0.081 |
| Depression | ATAI Use (per SD) | 1.00 | 0.83 | -0.64, 2.63 | 0.231 | 0.231 |
| Anxiety | AI a few times/week | 3.83 | 1.88 | 0.12, 7.55 | 0.043 | 0.081 |
| Anxiety | AI daily | 3.22 | 1.74 | -0.21, 6.65 | 0.066 | 0.098 |
| Anxiety | ATAI Benefit (per SD) | -2.39 | 0.77 | -3.91, -0.87 | 0.002 | 0.032 |
| Anxiety | ATAI Risk (per SD) | 1.44 | 0.71 | 0.04, 2.83 | 0.043 | 0.081 |
| Anxiety | ATAI Use (per SD) | 1.86 | 0.78 | 0.32, 3.40 | 0.018 | 0.081 |
| Stress | AI a few times/week | 3.55 | 2.21 | -0.81, 7.91 | 0.110 | 0.138 |
| Stress | AI daily | 3.24 | 2.19 | -1.09, 7.56 | 0.142 | 0.163 |
| Stress | ATAI Benefit (per SD) | -1.07 | 0.80 | -2.65, 0.50 | 0.181 | 0.194 |
| Stress | ATAI Risk (per SD) | 1.54 | 0.80 | -0.04, 3.11 | 0.056 | 0.093 |
| Stress | ATAI Use (per SD) | 1.84 | 0.83 | 0.20, 3.47 | 0.028 | 0.081 |

Note. Outcomes are doubled seven-item Depression, Anxiety and Stress scores (range 0–42 each). Every model uses the same 248 participants, covariates, reference groups and ATAI standardization as the total-score model. The models adjust for training year, sex, previous psychiatric treatment, AI frequency and all three ATAI factors. B is the adjusted change in the doubled subscale score. Nominal p-values and 95% CIs use HC3 standard errors and 235 residual degrees of freedom. Additional q-values use Benjamini–Hochberg correction across 15 focal final-model tests (two frequency contrasts plus three ATAI terms for each of the three subscales). This family is separate from the original three ATAI tests for DASS-21 total. Only the inverse Benefit–Anxiety association has additional q<.05. The estimates are exploratory, and similar directions across correlated outcomes are not independent replication.

# Supplementary Table S10 (continued). B. Subscale model fit and tests

| **Outcome** | **Block** | **R²** | **Adjusted R²** | **Change in R²** | **F-change (df₁,df₂)** | **p** | **HC3 added-block F (df₁,df₂)** | **p** | **HC3 overall F (df₁,df₂)** | **p** |
| --- | --- | --- | --- | --- | --- | --- | --- | --- | --- | --- |
| Depression | 1 | 0.080 | 0.054 | 0.080 | 3.00 (7,240) | 0.005 | 2.95 (7,240) | 0.006 | 2.95 (7,240) | 0.006 |
| Depression | 2 | 0.096 | 0.061 | 0.015 | 2.00 (2,238) | 0.137 | 2.30 (2,238) | 0.102 | 3.01 (9,238) | 0.002 |
| Depression | 3 | 0.140 | 0.096 | 0.044 | 4.02 (3,235) | 0.008 | 3.22 (3,235) | 0.023 | 3.28 (12,235) | <.001 |
| Anxiety | 1 | 0.123 | 0.097 | 0.123 | 4.79 (7,240) | <.001 | 4.26 (7,240) | <.001 | 4.26 (7,240) | <.001 |
| Anxiety | 2 | 0.134 | 0.102 | 0.012 | 1.59 (2,238) | 0.206 | 1.90 (2,238) | 0.152 | 3.89 (9,238) | <.001 |
| Anxiety | 3 | 0.198 | 0.157 | 0.063 | 6.19 (3,235) | <.001 | 4.80 (3,235) | 0.003 | 4.66 (12,235) | <.001 |
| Stress | 1 | 0.097 | 0.071 | 0.097 | 3.70 (7,240) | <.001 | 3.38 (7,240) | 0.002 | 3.38 (7,240) | 0.002 |
| Stress | 2 | 0.113 | 0.079 | 0.015 | 2.06 (2,238) | 0.130 | 1.89 (2,238) | 0.154 | 3.10 (9,238) | 0.002 |
| Stress | 3 | 0.150 | 0.106 | 0.037 | 3.41 (3,235) | 0.018 | 3.09 (3,235) | 0.028 | 2.93 (12,235) | <.001 |

Note. Block definitions and test interpretation are the same as in Table S9. Block 1 compares the covariate model with an intercept-only model; Block 2 tests added AI frequency; Block 3 tests added ATAI scores. Each outcome and step uses n=248. Parentheses give numerator and denominator degrees of freedom. The p-values in this panel are nominal block/model tests; the additional 15-test q-values apply only to the focal coefficients in panel A. Full coefficients, including all covariates and intercepts for every subscale and step, are available in Supplementary File 2, output 14.
